# Looking to the future of organs-on-chip

**DOI:** 10.4155/fsoa-2017-0040

**Published:** 2017-05-26

**Authors:** John Greenman

**Affiliations:** 1 School of Life Sciences, The University of Hull, Cottingham Road, Hull, HU6 7RX, UK

**Keywords:** ADME, lab-on-a-chip, microfluidics, organ-on-a-chip, tissue culture

The realization that immortalized cell lines cultured in 2D flasks do not fully reflect the *in vivo* behavior of an organ, is not a new concept; however, the ability to reconstruct organ complexity or maintain organ biopsies has not been available until recently [1]. In the last 20 years, lab-on-a-chip (LOAC) technology has become a reality, where the expertise from computer industry of fabricating microprocessors has been applied to the miniaturization of biological systems. Thus, microliter volumes of fluids can be accurately flowed over specific areas of a chip that can contain precisely patterned arrangements of cells, engineered tissue or patient biopsies. An important facet of this marriage of disciplines is that analytical systems can also be reduced to a similar scale so they too can be integrated onto the same platform where cells, spheroids or tissue biopsies are cultured [2], allowing accurate and precise determination of metabolites or biomarkers of interest on a miniature scale. So, although the number of cells or soluble analyte produced is low, the actual concentration within the device is sufficiently high for accurate and precise determination.

Prior to 2012, the biomedical applications of LOAC resided mainly within academic research laboratories and much innovative work continues there; however, with the National Institute of Health’s partnership with the Defense Advanced Research Projects Agency and the US FDA there was a major acceleration and step change. The initial investment of $13 million, part of a $70 million 5-year plan, supported 17 projects to both develop the technology for maintaining tissue and reconstruct one or more organs. The principal aim of this initiative was to provide new models that could be used for screening drugs in a cost-effective manner and thereby reduce the high drop-out and overall drug pipeline costs [3]. An additional advantage of the organ-on-a-chip (OOAC) approach is that it has the potential to at least reduce and in some instances replace animal models. The UK’s National Centre for Replacement, Refinement & Reduction of Animals in Research (NC3Rs) is one of the leading international scientific organizations supporting the development of alternative technology platforms with this avowed aim while pioneering better science [4]. Over the past decade, the devices have become more robust and currently several pharmaceutical companies are trialing devices [5]; also, there has been a drive to develop the LOAC approach to aid in stratifying patient responses to various therapeutic interventions [6].

In this special issue, colleagues have provided reviews of various applications of OOAC including work on stem cells, spheroids and tissue biopsies; there is novel research using bespoke devices for maintaining tumor biopsies as well as a user survey and two perspectives giving expert insight into the future of the OOAC field.

First, the review by Zhang and colleagues focuses on the use of embryonic stem cells and reprogrammed human-induced pluripotent stem cells and how these cells can be differentiated on chip to form organs [7]. This article clearly highlights the complexity of *in vivo* tissues, with relatively minor treatments of scaffolds or polymers resulting in major differences in organ formation and function. Following logically from the generation of organs, the article by Rogal and colleagues has reviewed the multiorgan field which has developed significantly since the big NIH-led investment in 2012 [8]. The approach that appears to have the most potential is the 'mix-and-match' model, where functional independent organ units are joined in a serial arrangement so that for instance, a drug metabolized by an on-chip liver can be transferred to a target organ, such as lung cancer to assess toxicity, or alternatively to the gut or brain, to allow assessment of off-target affects. The review by Ugolini and colleagues clearly illustrates that the field of OOAC is not limited to oncology as they discuss conditions and devices capable of maintaining cardiac cells and tissues [9]. Many similarities exist between the devices used by different researchers in terms of materials, channel sizes and flow rates; however, additional complexity is required when using cardiac tissue with a need for electrical stimulation and maintenance of tissue tension. The requirement to mimic the intrinsic complexity of *in vivo* cardiac tissue has in my opinion resulted in on-chip heart research falling a little behind that of the oncology applications.

The reviews herein highlight the potential use of OOAC devices by the pharmaceutical industry in drug development. The fourth article by Eva-Marie Dehne and colleagues entitled, “*The ascendance of microphysiological systems to solve the drug testing dilemma*” looks at the topic from an industry perspective and importantly highlights the role that regulatory bodies play [10]; as any shift to a new technology will require full acceptance by a number of national and international bodies [11]. The review particularly discusses how the microfluidic platforms address the ADME (absorption, distribution, metabolism and excretion) criteria that are core to the pharmacology community. Furthermore, the article by Huang reports the results of a survey of scientists working in pharma and academia and discusses what is expected of OOAC devices, in terms of the level of complexity which is deemed essential and what is desirable [12]. Despite concerns about reliability of these devices, one highly encouraging message is the general willingness of researchers from pharma and academia to adopt new models.

The article by Van Gent and colleagues describes the use of organotypic cultures and focuses on how these can be used in a high-throughput setting for drug screening [13]. A key point this article makes is that tissue slices are the best model of maintaining intratumoral heterogeneity and tumor–stromal interactions; also there is a significant benefit of being able to predict response in days/weeks that can then be used to inform clinical decisions. Another point that Van Gent makes is the need for standardization of approach, a point also highly relevant to regulatory bodies. The work by my own group has focused on the optimization of patient-derived tissue maintained in reliable and robust microfluidic devices for use in customizing therapy [14]. The paper first describes the modeling of fluid flow over the biopsy and ways of improving perfusion through modifications to the device, and secondly reports the importance of measuring cell viability as this is often not reflected in the tissue architecture.

Finally, this special edition contains two perspectives from Alexander Mosig, a life scientist [15] and John Wikswo, a biological physicist [16]. Despite coming from very different backgrounds there is a great deal of commonality in their respective views; both highlight that integration of systems is essential for effective replication of the *in vivo* environment and also acknowledge that this remains a challenge. Finally, John Wikswo said in an answer to the question “If you had unlimited funding, what would you do with it to further OOAC research?”, to which he replied “… What I would try to do is launch a program of intense characterization of the organs to try to understand the extent to which the *in vitro* cells on plastic and OOACs in humans and animal replicate real physiology”. I agree wholeheartedly that there needs to be a comparison of technologies to provide a rational basis on which researchers and end-users can select particular models.
